# Supplementary material for: Using a learning health system framework to examine COVID-19 pandemic planning and response at a Canadian Health Centre
Source: PLoS One. 2022 Sep 14;17(9):e0273149. doi: 10.1371/journal.pone.0273149 (PMC9473619; doi:10.1371/journal.pone.0273149)
Supplement: S1 File — (DOCX) [file pone.0273149.s001.docx]

Supplemental File 1. LHS Framework Matrix.

| **LHS Characteristic** | **Findings from Interviews, Administrative Data, and Textual Sources** | **Health System Receptors and Supports** | **Research System Supports** |
| --- | --- | --- | --- |
| **Engaged patients**: Systems are anchored on patient needs, perspectives and aspirations (at all levels) and focused on improving their care experiences and health at manageable per capita costs and with positive provider experiences | 1. Resource explaining to patients what the COVID-19 pandemic assessment centres (PAC) are like and what to expect when getting tested 2. Social media platforms (Facebook, Twitter, Instagram) and the Health Centre website were the main avenues of communication with public regarding cancellations, closures, reopening of services and visitor restrictions 3. Additional food services were made available due to the closures 4. Health Centre charities shifted its mandate to provide meals to support people 5. Health Centre COVID-19 Patient Survey was conducted to gather information of patient experience during the pandemic response 6. Patients described a lack of engagement in decisions related to closures of the Health Centre 7. Patients described a shift from patient-centred care as a result of the pandemic response 8. Patients described being well supported in terms of their access to personal protective equipment (PPE) 9. Patients described varying experiences due to visitor restrictions 10. Patients described that changes to food services did not support their needs 11. Staff described how changes in food services had a negative effect on patient experience 12. Staff were conflicted at having to implement visitor restrictions that were not aligned with the Health Centre’s mandate of patient centered care 13. Staff were challenged by managing both internal and external communication around visitor restrictions | **Assets:**   - The Developmental Clinic team developed resources for patients to better prepare them for the PAC - Communications & Public Affairs division   - Disseminated PAC information on Facebook   - Were responsible for all external messaging to the public - In the moment direct feedback (patients and families to front line HCP to managers) - Infection Prevent and Control (IPAC) Canada developed recommendations to ensure that patients could continue to access their belongings from home while also maintaining pandemic measures - Health Centre feedback services include   - ED experience surveys   - Experience with inpatient care survey   - Outpatient collection lab survey   - Pediatric Cancer Care – Outpatient survey   - Nurse/Dietician Allergy clinic survey   - Health Centre feedback email address   - Health Centre feedback phone line - Food Services & Business Development and IPAC collaborated on decisions regarding food services - Patient navigator role was developed to help patients navigate the Health Centre and access food   **Gaps:**   - Resources for patients about the PAC were only available through Facebook and not distributed through other social media platforms. - As the Health Centre transitioned into the reopening phase there was limited communication to the public through social media about changes in services - Health Centre patient engagement services (e.g., Patient Advisors, Youth Advisory Council, The Patient and Family Leadership Committee) were not utilized to help support the pandemic response - The burden of message development is left to individual departments, who do not always have adequate capacity to generate messaging while also accomplishing their regular task - No mechanism to support tailored communication to patients and families - Supports for support persons who stay long term | **Assets**:   - Health Centre Patient Survey   **Gaps:**   - Health Centre has a robust research network that was not used in the early phases of the pandemic to address issues related to engaging patients. Supports to facilitate greater communication between the leadership and research support could allow for the Health Centre research network to leverage its resources and help address issues which may arise as a result of the pandemic |
|  |  |  |  |
| **Digital capture, linkage and timely sharing of relevant data**: Systems capture, link and share (with individuals at all levels) data (from real-life, not ideal conditions) about patient experiences (with services, transitions and longitudinally) and provider engagement alongside data about other process indicators (e.g., clinical encounters and costs) and outcome indicators (e.g., health status) | 1. Administrative data related to the PAC were gathered    1. PAC volumes through the emergency department (ED)    2. Number of registrations at the PAC 2. Staff described the capability to track and share relevant staff data to facilitate redeployment to the PAC or other areas 3. Health Centre Dashboards 4. Epidemiological updates from Public Health were sent to leadership to help inform decision making 5. Scorecards with patient census data and waitlist profiles were sent out to leadership to help inform decision making 6. The Health Centre COVID-19 PPE Inventory Projection Model 7. Dedicated work from home (WFH) email set up to respond to any IT related issues staff had during the transition from WFH 8. Patients described varying levels of effectiveness of the Health Centre’s approach to information sharing 9. Staff described a lack of data capture in certain areas 10. Staff described that they initially lacked the ability to properly track PPE usage and stock 11. Staff reported that the feedback line allowed them to capture data from patients’ experiences of visitor restrictions | **Assets:**   - Leadership teams used PAC administrative data to inform decisions regarding redeployment to PAC, required capacity and changes in service hours - Health Centre PAC staff shared their expertise and resources with Provincial Health Authority colleagues - The Health Centre and Provincial Health Authority Performance and Analytics Teams collaborated to develop the COVID-19 Dashboards - Department of Health and Wellness (DHW) developed a COVID-19 Epidemiological Model which the Health Centre operationalized into hospital demand and capacity - The Strategy & Organizational Performance team   - Developed the Health Centre COVID-19 PPE Inventory Projection Model as a result of increased PPE usage, initial low storage volumes and unstable global supply chains.   - Used patient census data and waitlist data to develop scorecards - Health Centre COVID-19 subsite was instrumental in linking staff to up to date and relevant information regarding the evolution of the pandemic - Mental Health and Addictions had the capacity to gather data related to patient services usage changes and there are plans to use this to inform future service-related decisions - The Logistics and Resources Committee   - Led the development of allotment and auditing measures   - Coordinated all major issues regarding PPE sourcing, supplying and modeling - HR Department gathered data related to changes in staffing as a result of COVID-19 - Individual Departments were charged with tracking devices which were taken home. This data was sent to the IT Department   **Gaps:**   - Supports to enable sharing of PAC patient data between the Health Centre and other NS PACs. - Supports for analysing HR data captured during initial response - Supports to identify the availability of office space - Supports to capture volume of calls for dial for dining and data | **Gaps:**   - Need supports to link Health Centre research analytical capacity to HR data |
|  |  |  |  |
| **Timely production of research evidence**: Systems produce, synthesize, curate and share (with individuals at all levels) research about problems, improvement options and implementation considerations | 1. Seven COVID-19 studies were launched as part of the COVID-19 Health Research Coalition 2. Research Services modified their REB approval process to expedite COVID-19 related studies 3. Employee and physician survey aimed to explore 4. The impact of the COVID-19 pandemic on staff and physicians 5. Areas of success and areas of improvement of the Health Centre response 6. The level of staff and physician resilience and readiness for a potential second wave 7. Patients described a lack of awareness of evidence used to inform newly implemented pandemic restrictions | **Assets:**   - Members from Communications, Engagement & Change, People & Organization Development and Trauma Informed Care Departments collaborated with two leading workplace survey firms to design staff and physician survey - The results of the staff and physician survey were provided to the Executive Leadership Team and COVID-19 Leadership team to inform decision-making related to the COVID-19 response   **Gaps:**   - Supports to connect and allow different departments to leverage Health Centre Research Services resources (e.g., helping with rapid reviews of COVID-19 literature, understanding and critiquing the literature) | **Assets:**   - Provincial COVID-19 Health Research Coalition, collaborative partnership to develop a COVID-19 response strategy, supported research community with a commitment of $1.5 million - Mechanism for rapid REB review of COVID-19 studies   **Gaps:**   - Mechanisms for timely sharing of research evidence to support practice and policy change |
|  |  |  |  |
| **Appropriate decision supports**: Systems support informed decision-making at all levels with appropriate data, evidence, and decision-making frameworks | 1. Decisions concerning the PAC (i.e., screening, testing and the creation of the PAC) were supported by directives given by DHW and Provincial Health Authority. 2. Clinical Directives 3. Clinical Encounter Supports (IPAC) 4. Occupational Health and Wellness Supports 5. Human Resource Supports 6. Lab and Diagnostic Imaging Supports 7. Virtual Care Supports 8. Town Hall Q&A 9. Resources developed to support the transition to WFH 10. Informal professional health care provider networks 11. Weekly COVID-19 PPE Projection Reports 12. Contingency Plans 13. Patients described a lack of decision supports to guide them in decisions of when and when not to access care and whether it was safe to do so 14. Patients described inconsistencies in staff decision making related to enforcing visitor restriction 15. Staff described using alternate communication channels to access decision supports 16. Staff described the importance of having decision supports in place | - The Health Centre is a member of key working groups set up by the DHW through Public Health with the office of the Medical Officer of Health which guided provincial health system readiness - Decisions by staff and leadership are guided by their professional code of conduct and the Health Centre Ethics Framework and Strategy - The Logistics and Resources committee worked with IPAC as well as Provincial Health Authority and DHW to monitor, manage and maintain stock levels of essential supplies - The Strategy & Organizational Performance team began releasing weekly COVID-19 PPE Projection Reports - COVID-19 response leadership team coordinated decisions related to WFH policy changes   - The IT ticket system data helped informed decisions regarding change in IT policy - The People & Organization Development division developed WFH guidelines - WFH guidelines are available through the COVID-19 subsite   **Gaps:**   - The Health Centre Pandemic Response Plan was not used as it contained high level suggestions which did not cover the full breadth of the response. - Supports to enable ethical reviews of decisions and directives related to the pandemic response | None identified |
|  |  |  |  |
| **Aligned governance, financial and delivery arrangements**: Systems adjust who can make what decisions (e.g., about joint learning priorities), how money flows and how the systems are organized and aligned to support rapid learning and improvement at all levels | 1. The Health Centre PAC was integrated into the provincial 811 network allowing referrals from 811 for families with children 2. Centralizing and standardizing program and organizational level booking, registration and other team level services 3. Consulting with Unions on changes related to staff redeployment 4. Shifting to a virtual care model with the help of Provincial Health Authority 5. Some staff described a shift in their work responsibilities due to the response 6. Several COVID-19 related cost centres were set up to track costs related to COVID-19    1. PAC cost centre    2. Pandemic Response Unite cost centre    3. COVID-19 general cost centre    4. Good Neighbor Protocol cost centre    5. Entrance Screening cost centre    6. Resumption of Services cost centre 7. Visitor restrictions aligned with Provincial Health Authority 8. Staff reported that shifting to a WFH model improved morale 9. Staff described a lack of consistency and alignment in regard to who was leading decision making in certain areas 10. Staff in Food Services described having difficulty responding to changes in leadership that were a result of the pandemic response | - The People and Technology committee   - Worked with Unions to facilitate the rapid staffing changes and redeployment brought about by the pandemic response   - Worked with Unions to ensure standardization of resources   - Developed a virtual reassignment centre to remain in line with physical distancing measures and accommodate the shifting needs of the Health Centre   - Worked with different departments to ensure staff were equipped to WFH - The Medical Services Insurance program worked to accommodate the need for increased COVID-19 testing - Health Centre worked with Provincial Health Authority to implement a consistent and aligned pandemic response and shift to virtual care - The Financial Services & Treasury Department shifted their service to a decentralized model - Provincial Digital Services made ruling that security of VPN was not sufficient and that a move to Virtual Desktop Imaging was required. IT support at Health Centre facilitated this change   - Provincial Digital Services is a provincial service that supports the Health Centre for IT services and security - CVWG/EOC/IMC identified the need for a WFH approval process, this was delegated to HR who developed an FAQ and guidelines   **Gaps:**   - Supports to help adapt Provincial Health Authority directives to fit the context of the Health Centre - Supports to facilitate greater interdepartmental communication and information sharing | None identified |
|  |  |  |  |
| **Culture of rapid learning and improvement**: Systems are stewarded at all levels by leaders committed to a culture of teamwork, collaboration and adaptability | 1. The demand for testing, the ever-looming presence of the pandemic threat and maintenance of proper IPAC protocols resulted in continued collaboration between Provincial Health Authority and the Health Centre departments, as well as internal efforts to improve efficiency in terms of screening, testing, laboratory protocols and resource allocation. 2. Shared *Reimagining and Resuming Service* Town Hall presentation with DHW and Provincial Health Authority to aligned and ensure appropriate resumption of services. 3. Principles for Learning Activity during Pandemic Response (modified because of the pandemic response)    1. Prioritizing professional development investments    2. Choosing professional development programs    3. Budget considerations 4. Mental Health and Addiction Services    1. Imagining that triaging may occur virtually in the future, or consults for remote locations may occur virtually 5. A Virtual Education Committee was created to explore and facilitate shift to virtual care 6. Encouraging staff to share feedback on their WFH experience via Health Centre’s intranet newsletter 7. Staff described a shift in both the way they delivered care and the speed at which they had to adjust and improve upon the newly implemented changed 8. Staff reported being committed to facilitate a smooth transition to WFH | - The Health Centre continued to work with Provincial Health Authority to fulfill and adjust to local and provincial testing demands - COVID Leadership Team and the Logistics and Resources Committee all collaborated on different facets of PAC operations to ensure they continued and improved - The Health Centre shared pandemic related evidence with Provincial Health Authority and DHW - The Health Centre was informed by evidence and decisions generated by Provincial Health Authority, DHW and PHAC. - The Learning Team at the Health Centre: responsible for building organizational capacity through centre wide learning and development programs and services. - Health Centre Leadership - Mental Health and Addiction Services focused on adapting to virtual care as appropriate for future state - COVID Leadership Team and the Logistics and Resources Committee identified the need to expedite improvements of Health Centre’s PPE modeling and ordering infrastructure and facilitated this rapid change and improvement - Firsthand reports of negative patient impacts that arose from the visitor restrictions were discussed at the COVID Leadership Team meetings   **Gaps:**   - Supports to develop greater capacity for the Health Centre’s intranet newsletter | None identified |
|  |  |  |  |
| **Competencies for rapid learning and improvement**: Systems are rapidly improved by teams at all levels who have the competencies needed to identify and characterize problems, design data- and evidence informed approaches (and learn from other comparable programs, organizations, regions, and sub-regional communities about proven approaches), implement these approaches, monitor their implementation, evaluate their impact, make further adjustments as needed, sustain proven approaches locally, and support their spread widely | 1. PAC underwent    1. Location changes to accommodate increased capacity and mitigate disruption to other services    2. Reduction in hours in response to changing volumes 2. Changes in screening and testing protocols in response to evolving evidence and knowledge of COVID-19 3. Maintenance of COVID-19 Readiness and Responsiveness through gradual reopening 4. PAC remained open for testing capacity 5. Rapid Closure of non-essential services 6. Shifting certain non-urgent care areas to virtual care    1. Non-Urgent Mental Health & Addiction Services 7. Space Reassignment to accommodate    1. Increased storage capacity    2. PRU, PAC    3. Splitting ED (respiratory/infectious illness and all other urgent needs) 8. Staff Redeployment    1. PAC, PRU, group for airway management    2. Long term care    3. Kids Help phone 9. Maintenance of COVID-19 Readiness and Responsiveness through gradual reopening    1. The ability to ramp up the PRU within an hour    2. Responding to emerging evidence on COVID-19 pathologies by creating clinical care pathways and messaging    3. Bed designation    4. Staff readiness 10. Several changes to the visitor restriction occurred over time due to emerging evidence, patient response and reduction in reported provincial COVID-19 case 11. The capacity of Food Services to coordinate and implement decision making with COVID Leadership Team and IPAC on changes related to food closures, the development of additional food services to fill the gaps and plans for reopening 12. Patients described experiencing varying levels of competencies in terms of the Health Centre’s ability to respond to changing circumstances and implement changes 13. Staff reported that while they had competency for rapid change there were consequences for the staff 14. Staff recognize the importance of maintaining and developing competency to accommodate rapid changes that could result from future crises 15. Staff described a shift in the rate of learning and improvement as a result of the pandemic | - Fear and uncertainty of the pandemic revealed organizational capacity for rapid change, learning and improvement. The pandemic response created a unified objective for the Health Centre which was enacted by all staff at all levels of the organization - The ability of the Health Centre leadership team to coordinate the initial response through   - Collaboration with provincial governing bodies   - The creation of new organizational bodies   - Leveraging existing teams to address and respond to unprecedented challenges - The Health Centre Leadership team developed the *Reimagining and Resuming Services Plan*, which is a commitment by the Health Centre Leadership team to shift operations back to normal functioning while remaining agile in their ability to re-implement COVID-19 restriction should the need arise - The Health Centre leadership team, COVID Leadership team, and the Logistics and Resources committee aligned their objectives to address the rapidly changing needs of the healthcare system as it shifted its operations in response to the pandemic - The Innovation Technology & Redevelopment division collaborated with IT services to facilitate the shift to WFH - The People & Organization Development in consultation with DHW and Provincial Health Authority decided to maintain WFH due to global uncertainty and the minimal effect on productivity the change brought about - Food Services & Business Development, COVID Leadership Team and IPAC collaborated on decisions regarding food services | None identified |
|  |  |  |  |
