# Supplementary material for: Using a learning health system framework to examine COVID-19 pandemic planning and response at a Canadian Health Centre
Source: PLoS One. 2022 Sep 14;17(9):e0273149. doi: 10.1371/journal.pone.0273149 (PMC9473619; doi:10.1371/journal.pone.0273149)
Supplement: Attachment — Submitted filename: PLOS One Review 05.16.2021.docx [file pone.0273149.s002.docx]

Thank you for allowing me the opportunity to review this manuscript. This manuscript examines the use of a learning health system framework to explore COVID-19 planning and response. This work is both timely and critical to improve future pandemic planning.

**ABSTRACT**

In the background, I would be cautious to use terminology such as “wave one”, as I am not sure epidemiologists have yet been able to come to a consensus regarding how this pandemic has presented regarding case load.

**INTRODUCTION**

Line 89: Again, I would be cautious using wave one. Although you have operationalized this as prior to August 2020, I feel this may introduce unneeded controversy. I would instead state something similar to “early response to the COVID-19 pandemic” and include “(up to August 2020”, unless you are able to cite a respected work to back this timeline.

In line 91, the authors state the goal of the paper. Lines 96 through 99 states the aims of the paper as three research questions. This feels a little disjointed. I would instead suggest stating something similar to; “the goal was/is achieved by addressing the following aims/research questions…” to be clear on what is expected in the results of the manuscript.

**METHODS**

**Study Design**

Overall, this section should be expanded to provide more transparency in how the study was conducted. This could be improved in the following ways:

1. State why a mixed methods approach was the best way to answer the research questions.

2. As there are several approaches to mixed methods designs (this one choosing Creswell), list what data is being collected and used (administrative and textual data and interviews), when, and how. There is no need to go into depth about the data sources as this is detailed later in the methods, but an overview/preview would be helpful for flow.

3. The authors may want to consider adding a figure. It is stated on line 105 that iterative cycles of data collection/analysis were preformed, but it is not clear when or how. A flow diagram or something similar may be helpful for the reader to visualize the process. In such a figure, cycles of iteration and timing of data collection/analysis may be better understood.

4. The learning health system has an apparent large influence over this study, yet there are no mentions of a LHS framework nor the parallels between the components assessed in this study and the LHS. To be clearer, the authors should expand more on the rigor/trustworthiness of their work in assuring it ties to an LHS “lens”. This may be done through a conceptual model or further explanation.

**Study Setting**

Please confirm if this is indeed a “single” center, or if there are multiple locations in the system. Would also be interesting to know the approximate patient capacity, if available.

**Data Sources**

*Administrative and textual sources*

This explanation of the administrative and textual sources remains unclear until the explanation under the “Data analysis” section. At a minimum, a statement should be made regarding the purpose of choosing this particular data source. To mitigate this confusion, the authors may elect to combine the “Data sources” and “Data analysis” sections into one. Regardless, a clearer understanding of how this data will be used to reach the goals of the research (which also may be more clearly outlined in an overall figure as suggested above) is needed.

As a minor comment, the explanation regarding single reviewer examination and data organization may be better suited in the data analysis section/explanation.

*Qualitative Interviews*

Line 132: Mention of wave 1 (if changes are made to this verbiage)

Although details may be found in other manuscripts, it is still important to provide some context into the other studies to provide clarity for this analysis. It is also somewhat unclear what was analyzed previously and what (if any *new* data were generated in a qualitative analysis for this study) was done in the current manuscript regarding the interviews/thematic analysis. Please make this clearer to allow for greater transparency.

I would suggest that the interview guide be included as a supplementary material/appendix.

**DATA ANALYSIS**

**Administrative and textual data sources**

Between this section and “Data triangulation”, I don’t feel that there is adequate explanation of how the grouping the documents into three categories helped to facilitate a mixed-methods analysis. Although later the authors describe the development of a matrix structure, the purpose of examining the documents is unclear. What, if anything, was extracted from these documents? If they were simply grouped according to pillars of infection prevention and control/federal COVID guidelines, why not simply use these as guidance during the qualitative analysis (especially if no new categories emerged)?

**Qualitative interviews**

Line 153 should be deleted.

Although a method (directed content analysis) is cited, further detail of this method should be included. This could be a numbered list or descriptive text, but should address the general steps of the analysis, how many coders there were, if inter-rater reliability was calculated/assessed, assurances of trustworthiness, etc.

**Data triangulation**

Line 167-168- “through a series of individual and group tasks”. Such as?

**RESULTS**

Line 183- Again, even though the results are published elsewhere, there still should be some information available in this manuscript to make it a standalone paper. Qualitative themes could potentially be referenced here or included as a supplementary material/appendix. The number of participants/participant roles would also be helpful.

The following information in the results and the discussion are well written and interesting. However, I am having a difficult time drawing parallels between Figure 2, the subheadings in the results, and the subheadings in the discussion. It appears as there are three different interpretations to the data. The authors should make some effort to reorganize/relabel these sections to help readers with interpretation. Disclosing the matrix in a figure might be helpful. Regardless, the results should succinctly report how the authors came to their LHS-based improvements for future pandemic response, and then elaborate on these ideas and make specific suggestions in the discussion. Based on the three concurrent manifestations of these improvements/findings, I cannot give specific instruction on the best approach.

**LIMITATIONS**

The authors do not appear to disclose any limitations relevant to their work. This should be completed. One major limitation I have noted is the use of a women’s/children’s hospital as the sole location for data collection. This sample may limit the applicability to other health settings.

**TYPOGRAPHICAL AND GRAMMATICAL REVISIONS**

Line 61: “for the need to develop of sustainable”- I believe “of” should be omitted.

Line 62: Times should be time

Line 76: Lavis et al. define- should be defines or defined

Line 108: REB should be spelled out

**OVERALL COMMENTS**

Overall, this manuscript reads well and is in an area of interest to the journal’s audience. The findings are interesting but would benefit from greater clarity in the methods and a reorganization of the findings and discussion. Also please make sure to disclose relevant information from the other two studies your team has conducted- although some information may be available in other manuscripts, having such information available in this manuscript is essential so that it may be viewed as a stand-alone study.

Again, thank you to the authors for conducting this timely and relevant work, and to the editorial team at PLOS ONE for inviting me to review this manuscript.
