# Supplementary material for: Using a learning health system framework to examine COVID-19 pandemic planning and response at a Canadian Health Centre
Source: PLoS One. 2022 Sep 14;17(9):e0273149. doi: 10.1371/journal.pone.0273149 (PMC9473619; doi:10.1371/journal.pone.0273149)
Supplement: Attachment — Submitted filename: Learning health systems (LHS)_Pandemic.docx [file pone.0273149.s003.docx]

Review for the paper : Using a Learning Health System Framework to Examine COVID-19 Pandemic Planning and Response

The authors state that the aim of this paper is to present a study examining the assets and gaps in health system pandemic planning and response during wave one of the COVID-19 pandemic through the lens of Learning health systems (LHS). Guided by an integrated knowledge translation approach, the authors used a triangulation matrix to translate the data from organizational documents, system performance data and qualitative interviews with a range of relevant stakeholders into a common matrix. This lead to the identification of six priority domains relevant to COVID-19 Pandemic Planning and Response. However, when reading further, the study only examines data from a Canadian healthcare center and not, as the first parts of the paper claims, the whole health system. The paper addresses an important topic regarding the pandemic preparedness by analyzing documents and interviewing stakeholders of a tertiary care centre. I believe however that the paper could be improved before publication and therefore some suggestions are proposed here.

Introduction.

(lines 51 to 63). I had some difficulties to follow the reasoning of the authors describing how the Canadian health system responded to the pandemic. For instance, it is not very clear for the reader how telemedicine and virtual care options for non-urgent care lead to changes, or how this relates to the decrease in the use of ED for non-Covid related urgent care. Further, the motives of fear and distress experienced by pregnant and families experienced needs more explanation.

Therefore “these challenges” (line 60) need further elaboration (which challenges precisely?).

Lines 90-92: why did the authors chose this tertiary health centre while the introduction seems to imply the goal to identify “assets and gaps in (whole) health system”? Does this imply that the identification of promising strategies are limited to the learnings from one type of tertiary centre aiming at a fraction of the whole population?

Lines 93-95: could the authors state in a few words what this “larger program of research” is about and how the current study fits into that program?

Methods

Line 106: can you precise ‘regularly’?

Line 108: REB: I am not familiar with this term. Please clarify.

Line 129: how were the stakeholders identified?

Line 133: Why was an implementation framework used to explore the beliefs and attitudes towards pandemic preparedness and response processes? In other words, could the authors clarify what the link is between implementation and preparedness and response processes? Could the authors maybe clarify what concepts underly preparedness and response processes.

Line 157: see previous comment about an implementation framework used for identification of preparedness and response.

Lines 162-163: what processes/criteria were used by the teams to define key pandemic response priorities?

Lines 167-168: could the authors clarify what is meant by “a series of individual and group tasks.”

Results

Overall remark: It is not clear to the reader which results are based on which source and therefore how much credibility to attribute to the results presented here.

Lines 183-184: Detailed results will be published elsewhere but could the authors provide an overview of the results here, especially if they are under preparation?

Line 197: can you really speak about “engaging patients” while the information seemed only to flow one way from the centre to patients and patients and visitors seemed to be passive receptors of this information while no efforts were reported to check whether the information was understood and deemed relevant.

Line 209: could the authors explain what “relevant Covid19 were” in this aspect as the tertiary health centre is also embedded in a whole system. Therefore the dynamics of the epidemic in the community can also affect the centre and vice versa and data from the community are also needed. There is some information provided in Lines 221-223 but it is unclear for the reader what data were used (e.g. primary and secondary care centres).

Lines 253-254: “teams adapted directives to meet the specific needs of the organization and its patient population”. What is precisely meant by patient population? Is there a specific proactive policy to identify and reach out for vulnerable populations who are registred at the centre (e.g. children or mothers living at home, requiring long-term care from specialist services, outside the epidemic and for whom distancing measures have meant a break in continuity of care)?

Lines 262: only “to respond to the fast-paced changes needed to curb the spread of the virus.”? Not also to respond to the changes resulting from the distancing measures?

Lines 264-266: it is not very clear for the reader how the needs of patients with special needs, of people with poor digital literacy were addressed. Virtual care shows that the population of patients is not homogenous and that the needs of specific groups need specific strategies.

Lines 270: some examples would be helpful for the reader to better understand how and in which areas fear and uncertainty facilitated organizational capacity for rapid learning and improvement (e.g. illustrating the data-driven response).

Discussion: Since the text mentions that “informal communication with trusted sources was identified as the most prevalent strategy for knowledge exchange during wave one” (line 237 in results) and seeing how informal communication was limited due to physical distancing measures: how was this issue tackled?

I did not see a part discussing strengths and limitations of the study? No references were made to standards for conducting mixed methods research.

References: I would recommend to list only references that are available for the audience, not “manuscripts in preparation”
