# Supplementary material for: Using a learning health system framework to examine COVID-19 pandemic planning and response at a Canadian Health Centre
Source: PLoS One. 2022 Sep 14;17(9):e0273149. doi: 10.1371/journal.pone.0273149 (PMC9473619; doi:10.1371/journal.pone.0273149)
Supplement: Attachment — Submitted filename: R1.LHS Manuscript - Response Letter.docx [file pone.0273149.s004.docx]

| **Reviewer Comment** | **Author Response** |
| --- | --- |
| (lines 51 to 63). I had some difficulties to follow the reasoning of the authors describing how the Canadian health system responded to the pandemic. For instance, it is not very clear for the reader how telemedicine and virtual care options for non-urgent care lead to changes, or how this relates to the decrease in the use of ED for non-Covid related urgent care. Further, the motives of fear and distress experienced by pregnant and families experienced needs more explanation.  Therefore “these challenges” (line 60) need further elaboration (which challenges precisely?). | Thank you for highlighting this lack of clarity. We revised the first paragraph in the introduction to highlight that health systems had to make rapid changes as a result of the pandemic. See lines p. 3, lines 77-83 |
| Lines 90-92: why did the authors chose this tertiary health centre while the introduction seems to imply the goal to identify “assets and gaps in (whole) health system”? Does this imply that the identification of promising strategies are limited to the learnings from one type of tertiary centre aiming at a fraction of the whole population? | We revised this section to include a definition of health systems from the World Health Organization. A health system can be broad, nation-wide system, or smaller organizations. As such, for the purpose of this paper, we use the term health system to describe a tertiary health centre that serves three provinces. See page 7 lines 293-297. |
| Lines 93-95: could the authors state in a few words what this “larger program of research” is about and how the current study fits into that program? | Throughout this iteration, we have clarified that this paper reports solely on **the data triangulation protocol, using a LHS framework to map study findings**. To further clarify how this current study fits into the larger project, we included a methods figure (Figure 1), and detailed triangulation results (Supplemental File 1) |
| **Methods** |  |
| Line 106: can you precise ‘regularly’? | Revised to clarify these meetings were bi-weekly |
| Line 108: REB: I am not familiar with this term. Please clarify. | Revised |
| Line 129: how were the stakeholders identified? | Stakeholder identification was an iterative process. With our IKT approach, there were several health system administrator partners on the research team. These administrators identified key individuals within the organization to meet. From there, these meetings led to snowball identification of other key stakeholders recommendations. |
| Line 133: Why was an implementation framework used to explore the beliefs and attitudes towards pandemic preparedness and response processes? In other words, could the authors clarify what the link is between implementation and preparedness and response processes? Could the authors maybe clarify what concepts underly preparedness and response processes. | This section focused on the qualitative arm of this study which reported on the implementation of pandemic responses; as such, we used an implementation framework to understand the barriers and facilitators to implementation. This is a key component of a process evaluation. As we have refocused this paper to describe the triangulation protocol, including mapping on the LHS framework, we have removed these specific details. |
| Line 157: see previous comment about an implementation framework used for identification of preparedness and response. |  |
| Lines 162-163: what processes/criteria were used by the teams to define key pandemic response priorities? | The pandemic response priorities were generated through the semi-structured qualitative interviews and inductive thematic analysis. These methods are described in our companion paper that details the qualitative methods. Detailed triangulation data are available in supplemental file 1. |
| Lines 167-168: could the authors clarify what is meant by “a series of individual and group tasks.” | We have included more details on LHS triangulation methods on pages 7-9. |
| **Results** |  |
| Overall remark: It is not clear to the reader which results are based on which source and therefore how much credibility to attribute to the results presented here. | Thank you for raising this important point. We agree that the initial manuscript lacked clarity on the focus of this paper versus the other phases of this larger study. Therefore, we reworked this manuscript to clarify the focus on mapping the findings onto a LHS framework using a data triangulation protocol. By describing the mapping process in detail, this manuscript highlights assets and gaps related to pandemic response and planning. Further, it also demonstrates how a LHS framework can be used to examine rapid change and integration of evidence into practice. We included a detailed supplemental file that includes the triangulation matrix results to provide greater clarity on the data for this phase of the project.  By removing much of the details about the previous studies, this current iteration is clearer and more focused on the LHS component of the project. |
| Lines 183-184: Detailed results will be published elsewhere but could the authors provide an overview of the results here, especially if they are under preparation? | We clarified that this paper reports on the data mapping stage of the larger mixed methods project. We included the detailed triangulation results in supplemental file 1. |
| Line 197: can you really speak about “engaging patients” while the information seemed only to flow one way from the centre to patients and patients and visitors seemed to be passive receptors of this information while no efforts were reported to check whether the information was understood and deemed relevant. | We removed this section to avoid confusion and enhance clarity. We expand on patient engagement in the results and discussion sections as a significant gap in the pandemic response because of what you have mentioned – patients passively received important information and were not involved in the dissemination process. |
| Line 209: could the authors explain what “relevant Covid19 were” in this aspect as the tertiary health centre is also embedded in a whole system. Therefore the dynamics of the epidemic in the community can also affect the centre and vice versa and data from the community are also needed. There is some information provided in Lines 221-223 but it is unclear for the reader what data were used (e.g. primary and secondary care centres). | We removed this section to avoid confusion and improve alignment with paper objectives. |
| Lines 253-254: “teams adapted directives to meet the specific needs of the organization and its patient population”. What is precisely meant by patient population? Is there a specific proactive policy to identify and reach out for vulnerable populations who are registred at the centre (e.g. children or mothers living at home, requiring long-term care from specialist services, outside the epidemic and for whom distancing measures have meant a break in continuity of care)? | We have included a definition of what we mean by patient population (children, women and youth) page 7, lines 290-297. Our study did not identify proactive policies to engage vulnerable population. |
| Lines 262: only “to respond to the fast-paced changes needed to curb the spread of the virus.”? Not also to respond to the changes resulting from the distancing measures? | We have provided examples to describe the fast-paced changes, including physical distancing measures, increased use of PPE, and visitation restrictions (page 13, lines 560-561). |
| Lines 264-266: it is not very clear for the reader how the needs of patients with special needs, of people with poor digital literacy were addressed. Virtual care shows that the population of patients is not homogenous and that the needs of specific groups need specific strategies. | Virtual care has transformed health service delivery throughout the pandemic; however, this was beyond the scope of this study to examine how people with poor digital literacy were addressed. |
| Lines 270: some examples would be helpful for the reader to better understand how and in which areas fear and uncertainty facilitated organizational capacity for rapid learning and improvement (e.g. illustrating the data-driven response). | We included several examples on page 14, lines 569-570. |
| Discussion |  |
| Since the text mentions that “informal communication with trusted sources was identified as the most prevalent strategy for knowledge exchange during wave one” (line 237 in results) and seeing how informal communication was limited due to physical distancing measures: how was this issue tackled? | The data did not delve into the impact of physical distancing measures on informal communication. Instead, it highlighted the lack of formal linkages between the healthcare and research communities. |
| I did not see a part discussing strengths and limitations of the study? No references were made to standards for conducting mixed methods research. | We included this in a separate section at the end of the discussion page 21-22, lines 744-757. |
| References: I would recommend to list only references that are available for the audience, not “manuscripts in preparation” | Thank you for this suggestion; we have referenced the companion paper that is currently under review. |
|  |  |
| **REVIEWER #2** |  |
| In the background, I would be cautious to use terminology such as “wave one”, as I am not sure epidemiologists have yet been able to come to a consensus regarding how this pandemic has presented regarding case load.  Line 89: Again, I would be cautious using wave one. Although you have operationalized this as prior to August 2020, I feel this may introduce unneeded controversy. I would instead state something similar to “early response to the COVID-19 pandemic” and include “(up to August 2020”, unless you are able to cite a respected work to back this timeline. | Thank you for making this important distinction. We have removed all mention of wave one and replaced it with “early stages of the pandemic” and clarified that our findings represent that state of the pandemic up to August 2020. |
| In line 91, the authors state the goal of the paper. Lines 96 through 99 states the aims of the paper as three research questions. This feels a little disjointed. I would instead suggest stating something similar to; “the goal was/is achieved by addressing the following aims/research questions…” to be clear on what is expected in the results of the manuscript. | We have completely revised this section to clarify that the aim of this paper is to examine the utility of a LHS framework for examining rapid learning and change during the COVID-19 pandemic. From there, we define 3 specific objectives related to that overall aim. |
| METHODS |  |
| **Study Design** |  |
| Overall, this section should be expanded to provide more transparency in how the study was conducted. This could be improved in the following ways: |  |
| 1. State why a mixed methods approach was the best way to answer the research questions. | We added more details about our mixed methods approach in the study design section and clarified why a mixed methods research approach was needed to fully understand the topic of study (page 5, lines 210-218). |
| 2. As there are several approaches to mixed methods designs (this one choosing Creswell), list what data is being collected and used (administrative and textual data and interviews), when, and how. There is no need to go into depth about the data sources as this is detailed later in the methods, but an overview/preview would be helpful for flow. | We provided a detailed mixed methods research figure to clarify the approach (Figure 1). |
| 3. The authors may want to consider adding a figure. It is stated on line 105 that iterative cycles of data collection/analysis were preformed, but it is not clear when or how. A flow diagram or something similar may be helpful for the reader to visualize the process. In such a figure, cycles of iteration and timing of data collection/analysis may be better understood. | Thank you for this suggestion. We provided a mixed methods research figure as a visualization aid for the reader (Figure 1). |
| 4. The learning health system has an apparent large influence over this study, yet there are no mentions of a LHS framework nor the parallels between the components assessed in this study and the LHS. To be clearer, the authors should expand more on the rigor/trustworthiness of their work in assuring it ties to an LHS “lens”. This may be done through a conceptual model or further explanation. | We agree that the initial version of this manuscript lacked clarify on the LHS, despite the aim of this paper is to report the LHS mapping in detail. As such, we have made significant changes to refocus the manuscript on the LHS framework and how we used it to map the study findings. We included a new section that details the LHS framework and how it can be used to identify assets and gaps in health systems. Further, we included the triangulation matrix that includes the results mapped onto the LHS characteristics. |
| **Study Setting:** Please confirm if this is indeed a “single” center, or if there are multiple locations in the system. Would also be interesting to know the approximate patient capacity, if available. | We added more details about the Health Centre and its alignment as a health system. We included patient capacity details for 2020/2021 (page 7, lines 292-297). |
| **Data Sources**  *Administrative and textual sources*  This explanation of the administrative and textual sources remains unclear until the explanation under the “Data analysis” section. At a minimum, a statement should be made regarding the purpose of choosing this particular data source. To mitigate this confusion, the authors may elect to combine the “Data sources” and “Data analysis” sections into one. Regardless, a clearer understanding of how this data will be used to reach the goals of the research (which also may be more clearly outlined in an overall figure as suggested above) is needed.  As a minor comment, the explanation regarding single reviewer examination and data organization may be better suited in the data analysis section/explanation. | After reflecting on all reviewers’ comments, we recognized that our initial manuscript lacked clarity and focus. As such, we made significant changes to refocus this manuscript on the LHS framework mapping using a triangulation protocol. We removed details about the quantitative and qualitative data collection and analysis. Instead, we focused the methods section on our data triangulation methods using the LHS framework. Further, we added a supplemental file that goes into great detail on the mapping results for this phase of the project. We feel these changes have helped to strengthen the focus on the paper and highlight the utility of using a LHS framework to understand health system change. |
| *Qualitative Interviews*  Line 132: Mention of wave 1 (if changes are made to this verbiage)  Although details may be found in other manuscripts, it is still important to provide some context into the other studies to provide clarity for this analysis. It is also somewhat unclear what was analyzed previously and what (if any *new* data were generated in a qualitative analysis for this study) was done in the current manuscript regarding the interviews/thematic analysis. Please make this clearer to allow for greater transparency.  I would suggest that the interview guide be included as a supplementary material/appendix. |  |
| **Administrative and textual data sources**  Between this section and “Data triangulation”, I don’t feel that there is adequate explanation of how the grouping the documents into three categories helped to facilitate a mixed-methods analysis. Although later the authors describe the development of a matrix structure, the purpose of examining the documents is unclear. What, if anything, was extracted from these documents? If they were simply grouped according to pillars of infection prevention and control/federal COVID guidelines, why not simply use these as guidance during the qualitative analysis (especially if no new categories emerged)? |  |
| **Qualitative interviews**  Line 153 should be deleted.  Although a method (directed content analysis) is cited, further detail of this method should be included. This could be a numbered list or descriptive text, but should address the general steps of the analysis, how many coders there were, if inter-rater reliability was calculated/assessed, assurances of trustworthiness, etc. |  |
| **Data triangulation**  Line 167-168- “through a series of individual and group tasks”. Such as? | To refocus this manuscript on the data triangulation process, we included more details on the data triangulation methods section page 7-9. |
| **Results** |  |
| Line 183- Again, even though the results are published elsewhere, there still should be some information available in this manuscript to make it a standalone paper. Qualitative themes could potentially be referenced here or included as a supplementary material/appendix. The number of participants/participant roles would also be helpful. | Similar to our response above, we included more details about the triangulation methods and included a table with the triangulation matrix results to ensure there is enough information available to make this a standalone paper. |
| The following information in the results and the discussion are well written and interesting. However, I am having a difficult time drawing parallels between Figure 2, the subheadings in the results, and the subheadings in the discussion. It appears as there are three different interpretations to the data. The authors should make some effort to reorganize/relabel these sections to help readers with interpretation. Disclosing the matrix in a figure might be helpful. Regardless, the results should succinctly report how the authors came to their LHS-based improvements for future pandemic response, and then elaborate on these ideas and make specific suggestions in the discussion. Based on the three concurrent manifestations of these improvements/findings, I cannot give specific instruction on the best approach. | Thank you for highlighting this lack of clarity.  As described above, we made significant revisions to this manuscript to refocus on the data triangulation process and LHS framework mapping. As such, we removed mention of the qualitative and quantitative strand findings as the Results section of this manuscript should focus on the results of the mapping exercise. By making these changes, we feel the manuscript reads more clearly with the following section:  1. LHS framework mapping and triangulation **methods** (LHS framework details, triangulation protocol details);  2. Triangulation **results** (the findings from the quantitative and qualitative study mapped onto the seven LHS characteristics and identification of assets and gaps in the COVID-19 pandemic planning and preparedness work);  3. **Discussion** of the key findings related to assets and gaps, and the utility of the LHS framework to examine pandemic response (aligns with 3 main objectives) |
| **LIMITATIONS**  The authors do not appear to disclose any limitations relevant to their work. This should be completed. One major limitation I have noted is the use of a women’s/children’s hospital as the sole location for data collection. This sample may limit the applicability to other health settings. | We added a separate strengths and limitations section on page 21-22, lines 744-757. |
| **TYPOGRAPHICAL AND GRAMMATICAL REVISIONS**  Line 61: “for the need to develop of sustainable”- I believe “of” should be omitted.  Line 62: Times should be time  Line 76: Lavis et al. define- should be defines or defined  Line 108: REB should be spelled out | Thank you for highlighting these errors – all have been revised. |
