# Supplementary material for: Using a learning health system framework to examine COVID-19 pandemic planning and response at a Canadian Health Centre
Source: PLoS One. 2022 Sep 14;17(9):e0273149. doi: 10.1371/journal.pone.0273149 (PMC9473619; doi:10.1371/journal.pone.0273149)
Supplement: Attachment — Submitted filename: R2.Response to Reviewers.docx [file pone.0273149.s005.docx]

| **Comments** | **Response** |
| --- | --- |
| **Reviewer #3** |  |
| the paper is extremely vague on the methodology. It claims to use a mixed methods approach but it is very hard to get a sense of the nature or volume of data or sampling logic on the either the qual or quant part (how many interviews? what was this "health administrative and human resource data"?) | We appreciate the reviewers’ comments on the lack of description on our methodology. It is evident that we have not clearly articulated how this data triangulation papers builds on of previously collected and analyzed quantitative and qualitative data from an earlier stage of a larger research study. While we want to avoid restating the previous data collection and analysis, we recognize the need for further description and transparency of that stage of the research process.  As such, we added a table that describes the data collected in the quantitative and qualitative arms of the larger study. This table, along with Supplementary File 1 (Results table), provides more details on the data collection and results from the larger research study. From there, this current paper applies a LHS lens to the findings to describe how the health centre operates as a LHS in a health system crisis. See Table 2 on page 9. |
| the findings do not cross reference back to the data, and so it is difficult to judge the strength of the evidence based underlying the narrative. the findings often relate to things which were done (eg there was a covid subsite for sharing information) rather than rounded assessments of whether they worked. Many of the findings statements are very vague (eg "through the pandemic, systems shifted to align with national, provincial and local decisions and directives"; "the pandemic response created a unified objective for the health centre which was enacted by all staff at levels of the organization") and sound more like the self-congratulatory stories which managers tell themselves rather than true evidence-based research findings. | The purpose of the larger study was to *describe* the COVID-19 pandemic planning and response. As such, the team collected several sources of data that described different aspects of pandemic planning and response that occurred during an 8-month period. It was beyond the scope of this study to assess whether the actions worked. Instead, the aim was to describe what was happening, in real time, apply a LHS lens to understand assets and gaps, and inform the next stages of pandemic planning and response.  Now that we are two and a half years into the pandemic, it would be a valuable next step to assess how these pandemic measures, and changes made based on this study, worked and influenced patient health and health system outcomes. We described this in the limitations section and highlight this as a potential next step for future research and pandemic planning (page 22 lines 453-458). |
| The discussion does not clearly align with the findings. Partly this is a problem of structure: Given that the LHS concept has 7 dimensions, I would expect that the discussion would be organised along similar lines, but some of the subheadings relate to some dimensions and others do not. But it is also a problem of content: for example the section on EHRs gives reasons why EHRs are a good thing but these are reasons which could have been given without doing this study. | Thank you for this suggestion. We re-arranged the discussion into three sections to align with our research objectives.   1. First, we outline key unexpected findings within 3 of the LHS dimensions. We felt it was repetitive to go through the 7 dimensions again, having just stated them in the Results section. Therefore, we highlighted the most important discussion points within three dimensions. 2. Second, we highlight key findings that do not relate to the LHS dimensions. For example, an ethical framework for LHS is not a LHS dimension but our study illustrates why it is needed. This is an important study implication for future research and practice in LHS. We have highlighted this as an implication for moving the field of LHS forward on page 22, lines 417-419 3. Lastly, our application of a LHS framework to guide analysis of assets and gaps in a health care organization is novel. As such, it is important to discuss how it was used and implications for future application and development of LHS frameworks, tools, implementation strategies and evaluation metrics. This is described in the third section of the discussion. |
| A major weakness of the study design is that lack of a comparator. Ultimately all organisations are constrained by resources and structural factors. It's very hard to assess whether the current study setting did well or badly on the LHS dimensions without reference to what was done by other comparable entities which shared the same constraints. | The objectives of this study were to: i) map pandemic planning and response resources and strategies onto a LHS framework; ii) identify assets and gaps in the pandemic planning and preparedness work; and iii) describe how a LHS can be used as a framework to inform health system changes. As such, the purpose of the study was to describe the pandemic planning and preparedness using a LHS lens. A mixed methods research design was used to guide this work; it was beyond the scope of the study to evaluate the effectiveness of the LHS dimensions and compare to other organizations.  This comment speaks to the lack of evaluation of LHS literature. We highlighted this in the LHS implications and limitations section that further evaluative research is needed to compare organizations to test the effectiveness of the LHS dimensions on health system change. This descriptive study provides a strong foundation of which further effectiveness research can build upon. See page 23, lines 445-446 and page 24, lines 462-467. |
| Reviewer #4 |  |
| 1. While there is valuable learning from this study, particularly for other OECD countries, it is important to note that there is wide variation in the organisation, structure, and culture of health care systems and the application of the LHS approach may not be feasible or useful everywhere.  a. It should be clear earlier on in the manuscript that this is a single-site study at a Canadian hospital (e.g. in the title and abstract).  It would also be useful to provide some additional information for an international audience relating to the context of this healthcare system, e.g. insurance vs. publicly funded, rural/urban population served, key population-level demographic data for the area served such as socio-economic deprivation, age distribution, ethnicity | 1a. We added this to the title and abstract.  1b. We highlighted this as a limitation.  1c. We added more details on the Health Centre the settings section included how the health system is funded, and population demographic data on health status and income compared to the rest of Canada (page 7, lines 122-125) |
| 2. P. 5, line 102, fortnightly may be a better term than bi-weekly. | Revised |
| 3. P. 5, line 104, there is an additional full stop. | Revised |
| 4. P. 16, digital communication has been central to continuing provision of healthcare services during the pandemic, but consideration needs to be given to the potential impact of this on patient-centred care and to health inequalities, notably with regard to digital literacy and digital poverty. | This is an important point to consider when developing the digital infrastructure. We highlighted it as a key consideration in our recommendations. See page 20, lines 373-375 |
